# Hsa_circ_0088233 Alleviates Proliferation, Migration, and Invasion of Prostate Cancer by Targeting hsa-miR-185-3p

**DOI:** 10.3389/fcell.2020.528155

**Published:** 2020-10-30

**Authors:** Zhi-Hai Deng, Gan-Shen Yu, Ke-Lei Deng, Zhen-Hua Feng, Qiang Huang, Bin Pan, Jian-Zhong Deng

**Affiliations:** ^1^Department of Urology, Gaozhou People’s Hospital, Gaozhou, China; ^2^Department of Urology, Maoming People’s Hospital, Maoming, China; ^3^School of Medicine, Jiamusi University, Jiamusi, China; ^4^Department of Urology, The First Affiliated Hospital of Jinan University, Guangzhou, China

**Keywords:** circRNA, competitive endogenous RNA, invasion, migration, proliferation, prostate cancer

## Abstract

Prostate cancer is the most common malignant tumor of the urinary system. The mechanisms of the initiation and progression of prostate cancer have not been fully elucidated. Increasing evidence suggests that circular RNAs (circRNAs) are involved in cancer pathogenesis. In this study, we aimed to identify differentially expressed circRNAs in prostate cancer tissues and explored the role of circRNAs in the pathogenesis of prostate cancer. By screening a circRNA microarray assay, we found that circ_0088233 was upregulated in prostate cancer tissues compared to adjacent normal tissues, and this upregulation can be verified in 46 pairs of prostate cancer and adjacent normal tissues examined using quantitative reverse transcription-PCR. The level of circ_0088233 correlated with the TNM stage. Knockdown of circ_0088233 reduced cell proliferation, migration, invasion, and induced G1 phase arrest and apoptosis. In addition, miR-185-3p was identified as the downstream target of circ_0088233 using luciferase reporter assays and a biotinylated circ_0088233 probe pull-down assay. The miR-185-3p level showed a negative correlation with the circ_0088233 level in prostate cancer tissues. Overexpression of circ_0088233 blocked the effects of miR-185-3p on cell proliferation, migration, invasion, cell cycle, and apoptosis. In conclusion, circ_0088233 may function as an oncogene and play an oncogenic role by sponging hsa-miR-185-3p. This study increases the understanding of circRNAs in the progression of prostate cancer. These results implicate circ_0088233 as a potential therapeutic target for prostate cancer.

## Introduction

Prostate cancer is a malignant epithelial tumor of the prostate gland. It is the second most prevalent cancer worldwide (Bray et al., 2018). Prostate cancer is often prone to bone metastasis, resulting in bone pain or pathological fracture and paraplegia, and can be life-threatening. Patients with early prostate cancer can be treated with radical treatment. Moreover, patients with intermediate prostate cancer need comprehensive treatment, such as surgery combined with radiotherapy. Early detection, diagnosis, and treatment can lead to a good prognosis (Brody, 2015). Therefore, the identification of novel and practical biomarkers for prostate cancer diagnosis and target therapy is essential. Identification of such biomarkers will allow for the development of innovative targeted therapies to improve prostate cancer prognosis.

Circular RNAs (circRNAs) are a special type of non-coding RNA that have become the latest focus of RNA research (Chen and Huang, 2018; Vo et al., 2019). circRNAs are widely expressed in human cells. In contrast to traditional linear RNAs, which contain 5′ and 3′ termini, circRNA molecules have a closed, circular structure and do not affected by RNA exonuclease, therefore, their expression is relatively more stable, and they do not degrade easily (Meng et al., 2017). They have essential functions in regulating gene expression at the post-transcriptional level (Meng et al., 2017; Belousova et al., 2018). Recent studies have shown that circRNA molecules are rich in microRNA (miRNA)-binding sites and act as miRNA sponges in cells. circRNAs play a crucial regulatory role in diseases by alleviating miRNA-mediated inhibition of target genes and increasing the expression levels of these genes (i.e., competitive endogenous RNA, ceRNA) (Chen and Huang, 2018). Besides functioning as ceRNAs of miRNAs, some circRNAs play their roles in diseases by interacting with specific RNA-binding proteins (Kristensen et al., 2019). For instance, overexpression of circular RNA circFndc3b plays cardioprotective roles by modulating cardiac repair after myocardial infarction via the FUS/VEGF-A axis (Garikipati et al., 2019). Increasing evidence suggests that circRNAs play a role in cancer pathogenesis (Vo et al., 2019). At present, many researchers have reported that circRNAs can function as markers of prognosis and diagnosis of many cancer types such as prostate cancer (Tucker et al., 2020). Moreover, circRNAs regulate cancer cell growth, metastasis, and drug resistance (Liang et al., 2020; Ma et al., 2020).

This study aimed to identify differentially expressed circRNAs in prostate cancer tissues and explored the role of circRNAs in prostate cancer’s pathogenesis. These findings provide a better understanding of the role of circRNAs in prostate cancer and will inform the development of novel therapeutic strategies for prostate cancer.

## Materials and Methods

### Specimens

Prostate cancer tissues (*n* = 46) and adjacent normal tissues (*n* = 46) were obtained from Gaozhou People’s Hospital from 2017 to 2018. Informed consent was obtained from all patients according to standard procedures of the local ethics committee. This study received institutional review board approval and was carried out according to the regulations of Declaration of Helsinki.

### circRNA Microarray Assay

The expression profile of circRNAs in one pair of prostate cancer tissue sample and adjacent normal tissue from one patient (66 years old; tumor stage: III; lymphatic metastasis: No) was generated using the Human CircRNA Array v2 (Zhao et al., 2017) (CapitalBio Technology, Beijing, China) containing 170,340 human circRNA probes. The circRNA target sequences were all from Circbase and Deepbase. Each circRNA was simultaneously detected using a long probe and a short probe. The circRNA array data were analyzed for data summarization, normalization, and quality control using the GeneSpring software V13.0 (Agilent, Santa Clara, CA, United States). To select differentially expressed genes, we used threshold values of ≥2 and ≤2-fold-change and a Benjamini–Hochberg-corrected *P*-value of 0.05. The data were log2 transformed and median-centered by genes using the Adjust Data function of CLUSTER 3.0 software, and then further analyzed with hierarchical clustering with average linkage.

### Cell Culture

Human prostate cancer cells (22Rv1, Du145, LNCaP, and PC3) and RWPE-1 prostate epithelial cells were obtained from the American Type Culture Collection (Manassas, VA, United States). The 22RV1, Du145, LNCaP, and PC3 cells were grown in RPMI-1640 medium containing 10% fetal bovine serum (FBS), 100 U/mL penicillin G, and 100 μg/mL streptomycin at 37°C in a humidified 5% CO_2_ incubator.

### Construction of circ_0088233 Stable Knockdown Cells

Lentiviruses expressing short hairpin RNA (shRNA) targeting circ_0088233 (LV-sh-circ#1 and LV-sh-circ#2) and lentivirus expressing negative control shRNA (LV-sh-NC) were purchased from GenePharma (Shanghai, China). LNCaP and PC3 cells were infected with LV-sh-NC, LV-sh-circ#1, or LV-sh-circ#2 (multiplicity of infection = 50). After culturing in the presence of purinomycin for 3–4 weeks, the circ_0088233 level successfully decreased, as determined using quantitative reverse transcription-PCR (qRT-PCR). Cells infected with LV-sh-NC, LV-sh-circ#1, or LV-sh-circ#2 were termed sh-NC, sh-circ#1, or sh-circ#2 groups.

### circ_0088233 Overexpression Plasmid, miR-185-3p Mimic, and miR-185-3p Inhibitor Synthesis

Full-length circ_0088233 was cloned into the pLCDH-ciR circRNA overexpression plasmid (Guangzhou Geneseed Biotech Co., Ltd., Guangzhou, China), termed as pLCDH-ciR-circ_0088233. Negative control miRNA (miRNA), miR-185-3p mimic, inhibitor NC, and miR-185-3p inhibitor were synthesized by GenePharma (Shanghai, China). Lipofectamine 3000 (Invitrogen, Carlsbad, CA, United States) was used to transfect miRNA or plasmid.

### Cell Group

LNCaP and PC3 cells of sh-NC, sh-circ#1, and sh-circ#2 groups were used to investigate the role of circ_0088233. Moreover, LNCaP and PC3 cells were divided into four groups: miR-NC group (transfected with miR-NC), miR-185-3p group (transfected with miR-185-3p mimic), miR-185-3p + vector group (transfected with miR-185-3p mimic and empty vector pLCDH-ciR), and miR-185-3p + circ_0088233 group (transfected with miR-185-3p mimic and pLCDH-ciR-circ_008823). The division was made to investigate whether overexpression of circ_0088233 blocks the effects of miR-185-3p on cell proliferation, migration, invasion, cell cycle, and apoptosis.

### Quantitative Reverse Transcription PCR

Total RNA was extracted from tumors and normal tissues using TRIzol reagent (Ambion, Austin, TX, United States). After reverse transcription, cDNA was used as a template to determine circRNA and miRNA levels using SYBR GREEN qPCR Super Mix (Invitrogen) on an ABI PRISM 7500 system (Applied Biosystems, Foster City, CA, United States). Glyceraldehyde-3-phosphate dehydrogenase (*GAPDH*) was used to normalize circRNA expression levels. U6 was used to normalize the miRNA expression levels. The primers used are listed in Supplementary Table S1.

### Cell Proliferation Assay

Cell proliferation was analyzed using the CellTiter 96^®^ AQueous One Solution Cell Proliferation Assay kit (Promega, Madison, WI, United States). The cells were seeded in wells of 96-well plates. After seeding for 24, 48, and 72 h, 20 μL CellTiter 96^®^ AQueous One Solution Reagent was added to each well. The plates were incubated for 2 h, and the absorbance was measured at 490 nm using a microplate reader.

### Transwell Assays

Transwell assays evaluated the migration and invasion of LNCaP and PC3 cells. For the migration assay, cells from each group were digested with trypsin and washed with serum-free medium three times. Cell pellets were resuspended in serum-free medium, and 100 μL cell suspension (10,000 cells per well) were added into the upper chamber. Six hundred μL medium containing 20% FBS was added into the lower chamber. After incubation in a 37°C incubator for 24 h, the Transwell chamber was washed twice with PBS and fixed with 5% pentanediol at 4°C. After that, the cells were stained with 0.1% crystal violet solution for 10 min. After washing twice with PBS, the cells at the top of the filter membrane were removed using a cotton swab. The migrated or invading cells at the bottom of the filter membrane were monitored and photographed using a DMi8 microscope (Leica, Wetzlar, Germany) at 200 × magnification. Six randomly selected fields were counted. For invasion assay, the transwell chamber precoated with matrigel was used, and the experimental procedure was the same as the migration assay. Each experiment was repeated three times. The average cell number per field was used as the migrated or invading cell number.

### Flow Cytometry Analysis for Cell Cycle and Apoptosis

The cell cycle detection kit (Nanjing KeyGen Biotech Co. Ltd., China) was used for cell cycle analysis. Briefly, harvested cells were fixed with pre-cooled 70% ethanol overnight at 4°C. The next day, cells were washed with PBS and incubated with 500 μL propidium iodide staining solution containing 100 μg/ml RNase A and 0.2% Triton X-100 at 4°C for 30 min. After that, the cell cycle was analyzed using a FACSCalibur flow cytometer (BD Biosciences, San Jose, CA, United States), and the results were analyzed using Modfit LT version 2.0 (Verity Software House Inc., Topsham, ME, United States).

Annexin V-FITC/PI Apoptosis Detection Kit (Nanjing KeyGen) was used for apoptosis. Briefly, harvested cell pellets were resuspended in 500 μL of 1 × binding buffer. After that, 1.25 μL Annexin V-FITC solution was added to the cell suspension. After incubation for 15 min at 18–24°C, the cell suspension was centrifuged (1000 *g*, 5 min), and cell pellets were resuspended in 500 μL 1 × binding buffer. Propidium iodide (10 μL) was added to the cell suspension. Within 1 h, apoptosis was analyzed using a FACS caliber flow cytometer (BD Biosciences), and the results were analyzed using FlowJo software version 8 (FlowJo, Ashland, OR, United States).

### Subcutaneous Tumors Models in Nude Mice

Four - to six-week-old female nude mice were obtained from Beijing Vital River Laboratory Animal Technology Co. Ltd. (Beijing, China). Twenty-four nude mice were divided into four groups (*n* = 6). The mice were subcutaneously injected with 5 × 10^6^ of circ_0088233-stable LNCaP or PC3 cells or control cells in their dorsal flanks. Tumor volumes were tested 12, 14, 16, 18, 20, 22, 24, 26, 28, and 30 days after injection. All experimental procedures involving animals were approved by our Institutional Animal Care and Use Committee of Gaozhou People’s Hospital following the Guide for the Care and Use of Laboratory Animals published by the National Institute of Health (NIH Publication No. 85–23, revised 1996).

### Luciferase Reporter Assays

To create a luciferase reporter plasmid containing the linear sequence of circ_0088233, a full-length linear sequence of circ_0088233 was cloned into the dual-luciferase miRNA target expression vector GP-mirGLO (Promega). The plasmid was designated as wild type circ_0088233. The binding sites of miR-185-3p in wild-type circ_0088233 (located at 586–605 bp and 721–739 bp) were mutated using the Site-Directed Mutagenesis Kit (Stratagene, San Diego, CA, United States). The constructs were designated as circ_0088233 mutant 1 (binding site at 586–605 bp was mutated), circ_0088233 mutant 2 (binding sites at 721–739 bp were mutated), and circ_0088233 mutant 3 (binding site at 586–605 bp and 721–739 bp). The luciferase reporter plasmids were verified by sequencing. The primers used to amplify the linear sequence of circ_0088233 and create mutants are listed in Supplementary Table S2.

Human embryonic kidney 293T cells were seeded into wells of 96-well plates. Cells were co-transfected with the luciferase reporter vector and miR-185-3p mimic or miR-NC. After transfection for 48 h, luminescence was measured using Dual-Glo Luciferase Assay reagents (Promega) according to the manufacturer’s protocol. The experiments were conducted in triplicates.

### Biotinylated circ_0088233 Probe Pull-Down Assay

LNCaP and PC3 cells at 50% confluence (approximately 2 × 10^6^ cells) were transfected with 50 μM of biotinylated circ_0088233 probe (100 bp reverse complementary sequence of circ_0088233) or control probe (random sequence). At 24 h after transfection, the cells were harvested, and the pull-down assay was carried out as previously described (Phatak and Donahue, 2017). The levels of circ_0088233 and miR-185-3p in enriched RNAs were determined using qRT-PCR using the same method described above.

### Western Blot Analysis

Total proteins were extracted using RIPA Lysis Buffer and subjected to sodium dodecyl sulfate-polyacrylamide gel electrophoresis (SDS-PAGE). The proteins were transferred to polyvinylidene fluoride membranes. The membranes were blocked with 5% non-fat milk and then incubated with primary antibody (anti-WNT2B, anti-E2F1, or anti-GAPDH). After incubation with the secondary antibody, SuperSignal West Pico Plus (Thermo Fisher Scientific, Waltham, MA, United States) was used for protein visualization. The information for the primary antibody is shown below: anti-WNT2B antibody (ab203225, 1: 800), anti-E2F1 antibody (ab137415, 1:2000), and anti-GAPDH antibody (ab9485, 1:2500). All primary antibodies were purchased from Abcam (Cambridge, MA, United States).

### Statistical Analyses

The statistical analysis of circRNAs and miR-185-3p expression in prostate cancer tissues and adjacent normal tissues was performed using the Mann–Whitney test. The circ_0088233 and miR-185-3p expression results in prostate cancer tissues, and adjacent normal tissues are shown as the median (25% percentile, 75% percentile). The statistical analysis between the two groups (sh-NC vs. sh-circ#1; sh-NC vs. sh-circ#2; miR-NC vs. miR-185-3p; miR-185-3p + vector vs. miR-185-3p + circ_0088233) in functional assays in the cell lines and animal models were analyzed using an unpaired *t*-test. Linear regression analysis was carried out to analyze the correlation between miR-185-3p and circ_0088233 expression levels. All statistical analyses were performed using GraphPad Prism version 7.0 (GraphPad Software, San Diego, CA, United States). A *P*-value < 0.05 was considered significant.

## Results

### Expression Profiling of circRNAs in Prostate Cancer Tissues

Differentially expressed circRNAs in prostate cancer were identified using the circRNA array. All data were submitted to Gene Expression Omnibus (GEO) DataSets, and the GEO accession number is GSE155792^1^.

### Up-Regulation of circ_0088233 in Prostate Cancer Tissues and Cell Lines

**FIGURE 1:**
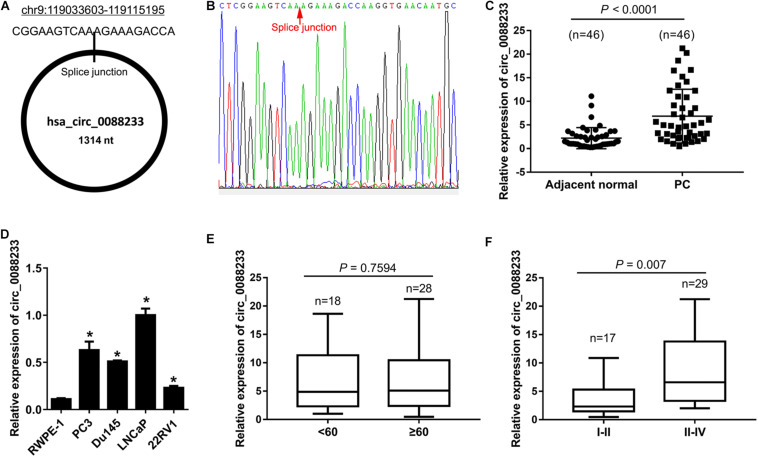
Upregulation of circ_0088233 in prostate cancer tissues and cell lines. **(A)** Location and length information of circ_0088233. **(B)** The sequence result of the circ_0088233 fragment in qRT-PCR. **(C)** Expression level of circ_0088233 in prostate cancer tissues (PC, *n* = 46) and adjacent normal tissues (*n* = 46). **(D)** Expression level of circ_0088233 in prostate cancer cell lines (22RV1, Du145, LNCaP, and PC3) and RWPE-1 prostate epithelial cells. **(E)** Expression level of circ_0088233 in PC tissue of patients < 60 and ≥60 years of age. **(F)** Expression level of circ_0088233 in PC tissue of TNM I-II patients and TNM III-IV patients. *P < 0.05, when compared to RWPE-1.

The expression levels of the top five upregulated circRNAs (circ_0043592, circ_0051240, circ_0053382, circ_0088220, and circ_0088233) in 12 prostate cancer tissues and 12 adjacent normal tissues were examined using qRT-PCR. As shown in Supplementary Figure S1, only circ_0088233 was significantly upregulated in prostate cancer tissues compared to adjacent normal tissues. Next, we further confirmed the circ_0088233 level in 46 pairs of prostate cancer and adjacent normal tissues. circ_0088233 was localized at chr9:119033603-119115195. Its spliced length was 1314 nt. The splice junction is shown in Figure 1A. The sequence result of the circ_0088233 fragment in qRT-PCR is shown in Figure 1B. As shown in Figure 1C, significant upregulation of circ_0088233 was identified in prostate cancer tissues (4.875 [2.318, 10.9]) compared to adjacent normal tissues (1.375 [0.8375, 2.968]). Also, circ_0088233 was upregulated in prostate cancer cell lines 22RV1, Du145, LNCaP, and PC3, compared to RWPE-1 prostate epithelial cells (Figure 1D).

The circ_0088233 level was not significantly different (*P* = 0.7594) in prostate cancer tissue of patients < 60 and ≥60 years of age (Figure 1E). Significant differences were evident in prostate cancer tissues of TNM I-II patients and TNM III-IV patients (*P* = 0.007), indicating that the circ_0088233 level was correlated with TNM stage (Figure 1F).

### Knockdown of circ_0088233 Reduces Cell Proliferation, Migration, and Invasion

**FIGURE 2:**
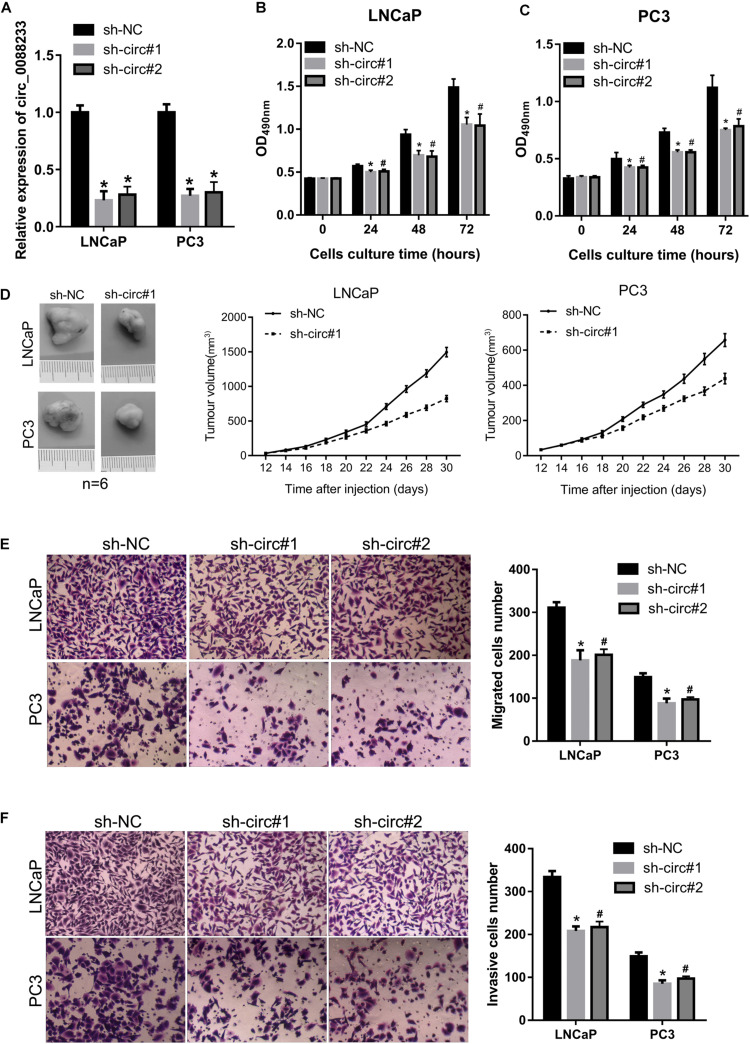
The knockdown of circ_0088233 reduces cell proliferation, migration, and invasion. LNCaP and PC3 cells featuring stable knockdown of circ_0088233 were constructed by infection with lentivirus expressing shRNA targeting circ_0088233 (sh-circ#1 and sh-circ#2). Cells infected with lentivirus expressing negative control shRNA (sh-NC) were used as control. **(A)** Levels of circ_0088233 in sh-NC, sh-circ#1, and sh-circ#2 cell groups determined using qRT-PCR. **(B,C)** Optical density at 490 nm (OD_490_) of sh-NC, sh-circ#1, and sh-circ#2 groups of cells. **(D)** The volume of subcutaneous tumors formed by sh-NC, sh-circ#1, and sh-circ#2 groups of cells. **(E,F)** Migrated **(E)** and invasive **(F)** numbers of sh-NC, sh-circ#1, and sh-circ#2 cell groups per field. *P < 0.05, when sh-NC vs. sh-circ#1; ^#^*P* < 0.05, when sh-NC vs. sh-circ#2.

As shown in Figure 1C, circ_0088233 levels were higher in LNCaP and PC3 cells. Therefore, these two cells were chosen for the functional assays. LNCaP and PC3 cells featuring stable knockdown of circ_0088233 were constructed. The results of qRT-PCR showed that circ_0088233 level was lowered by the two circ_0088233 shRNAs (sh-circ#1 and sh-circ#2) compared to sh-NC (Figure 2A). The effects of circ_0088233 knockdown on the proliferation, migration, and invasion of PC cells were then investigated. As shown in Figures 2B,C, the optical density at 490 nm of the sh-circ#1 and sh-circ#2 groups of cells was significantly lower than the sh-NC group. The volume of subcutaneous tumors formed by the sh-circ#1 group was significantly smaller than that formed by the sh-NC group (Figure 2D). The numbers of migrating and invading sh-circ#1 and sh-circ#2 cells were significantly less than the number of sh-NC cells (Figures 2E,F). These results suggested that circ_0088233 knockdown reduced cell proliferation, migration, and invasion abilities of LNCaP and PC3 cells.

### Knockdown of circ_0088233 Induces G1 Phase Arrest and Apoptosis

**FIGURE 3:**
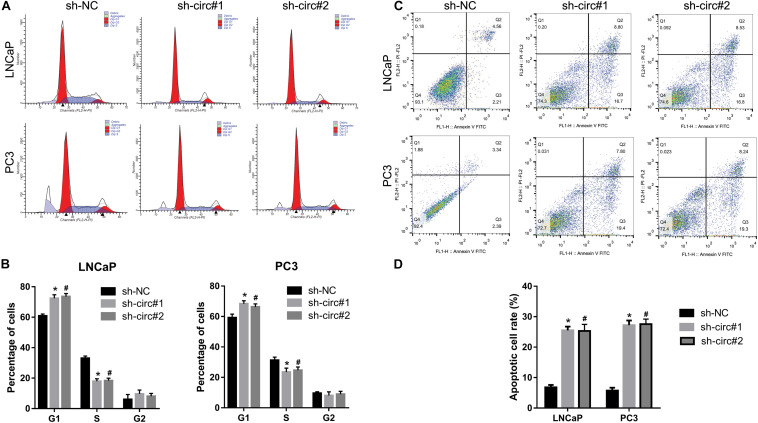
The knockdown of circ_0088233 induces G1 phase arrest and apoptosis. The percentage of cells in G1, S, and G2 phases **(A,B)** and apoptotic cells rate **(C,D)** were measured using flow cytometry analysis. LNCaP and PC3 cells featuring stable knockdown of circ_0088233 (sh-circ#1 and sh-circ#2) exhibited more cells in the G1 phase and higher apoptosis level compared to the negative control shRNA (sh-NC) group. **(A,C)** Are the representing images. **(B,D)** Are the statistical results of three independent assays. *P < 0.05, when sh-NC vs. sh-circ#1; ^#^*P* < 0.05, when sh-NC vs. sh-circ#2.

As shown in Figures 3A,B, the percentage of cells in the G1 phase was higher, and the percentage of cells in the S phase was lower in sh-circ#1 and sh-circ#2 groups compared to the sh-NC group. In addition, the percentage of apoptotic cells was higher in sh-circ#1 and sh-circ#2 groups than in the sh-NC group (Figures 3C,D). These results revealed that knockdown of circ_0088233 induced G1 phase arrest and apoptosis in LNCaP and PC3 cells.

### Binding of miR-185-3p to circ_0088233

**FIGURE 4:**
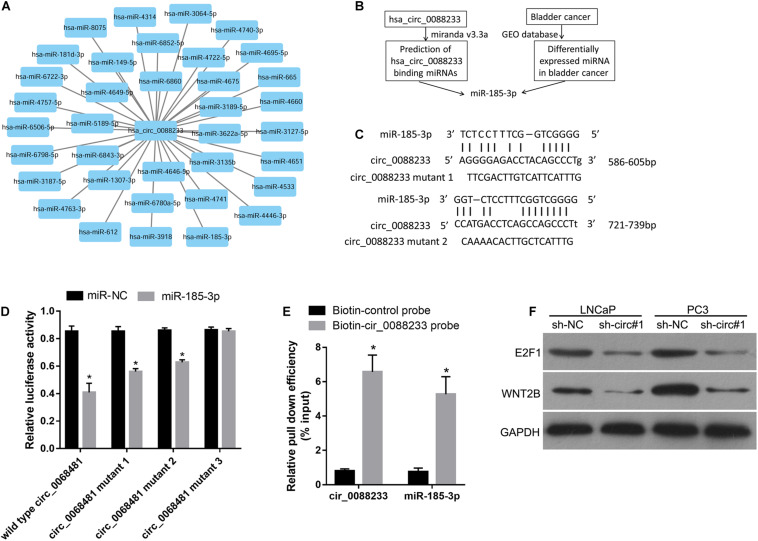
miR-185-3p can bind to circ_0088233. **(A)** Thirty-six miRNAs with binding sites on the circ_0088233 sequence. **(B)** Flow diagram showing how miR-185-3p was chosen. **(C)** The binding sites of miR-185-3p in the sequence of circ_0088233. **(D)** The effect of miR-NC and miR-185-3p mimic transfection on the relative luciferase activity of luciferase reporter vector containing a linear sequence of circ_0088233, linear sequence of circ_0088233 with one mutated miR-185-3p binding site (circ_0088233 mutant 1 and 2), or linear sequence of circ_0088233 with two mutated miR-185-3p binding sites (circ_0088233 mutant 3). *P < 0.05, when miR-NC vs. miR-185-3p. **(E)** The results of biotinylated circ_0088233 probe pull-down assay. The level of circ_0088233 and miR-185-3p was higher in RNAs enriched by biotinylated circ_0088233 probe compared to the biotinylated control probe. *P < 0.05, biotin-circ_0088233 probe vs. biotin-control probe. **(F)** Protein levels of miR-185-3p targets E2F1 and WNT2B in sh-circ#1 and sh-NC groups of cells.

The miRNA response elements were predicted based on the sequence of circ_0088233 to investigate its mechanism. As shown in Figure 4A, 36 miRNA response elements were identified. This finding indicated that circ_0088233 might sponge one or more miRNAs (Figure 4A) by the ceRNA mechanism to regulate the miRNA targets. As circ_0088233 knockdown resulted in reduced proliferation, migration, and invasion, we predicted that the target miRNA of circ_0088233 is a tumor suppressor downregulated in cancer. By screening the differentially expressed miRNAs in PC in the Gene Expression Omnibus database, we found that only miR-185-3p was downregulated (Figure 4B). This finding further confirmed the relationship between miR-185-3p and circ_0088233.

There were two miR-185-3p binding sites in circ_0088233 (Figure 4C). The dual-luciferase assay was performed to validate the binding of miR-185-3p to the linear sequence of circ_0088233. As shown in Figure 4D, the relative luciferase activity of miR-185-3p mimic and wild type circ_0088233 co-transfection group was decreased compared to the miR-NC and wild type circ_0088233 co-transfection group, indicating that miR-185-3p overexpression can suppress the relative luciferase activity of the luciferase reporter vector containing a linear sequence of circ_0088233 as the artificial 3′ untranslated region. Moreover, miR-185-3p overexpression also suppressed the luciferase reporter vector’s relative luciferase activity containing linear circ_0088233 with one mutated miR-185-3p binding site (circ_0088233 mutant 1 and 2) as the artificial 3′ untranslated region. However, miR-185-3p overexpression did not affect the luciferase reporter vector’s relative luciferase activity containing linear circ_0088233 with two mutated miR-185-3p binding sites (circ_0088233 mutant 3). These results suggested that miR-185-3p could bind to the linear sequence of circ_0088233. A biotinylated circ_0088233 probe pull-down assay was carried out to confirm whether miR-185-3p could bind to circ_0088233 in prostate cancer cells. As shown in Figure 4E, the levels of both circ_0088233 and miR-185-3p were higher in RNAs enriched with the biotinylated circ_0088233 probe compared to the biotinylated control probe, indicating that the circ_0088233 probe can pull down both circ_0088233 and miR-185-3p. These results suggested that miR-185-3p could bind to circ_0088233 in prostate cancer cells.

To further verify whether circ_0088233 as a ceRNA of miR-185-3p, we investigated its effect on the level of circ_0088233 knockdown on the levels of WNT2B and E2F1, which are the targets of miR-185-3p identified in cancer cells (Liu et al., 2017; Lu et al., 2018). As shown in Figure 4F, the protein levels of E2F1 and WNT2B were lower in the sh-circ#1 group than in the sh-NC group.

### Negative Correlation of miR-185-3p Level With circ_0088233 Level in PC Tissues

**FIGURE 5:**
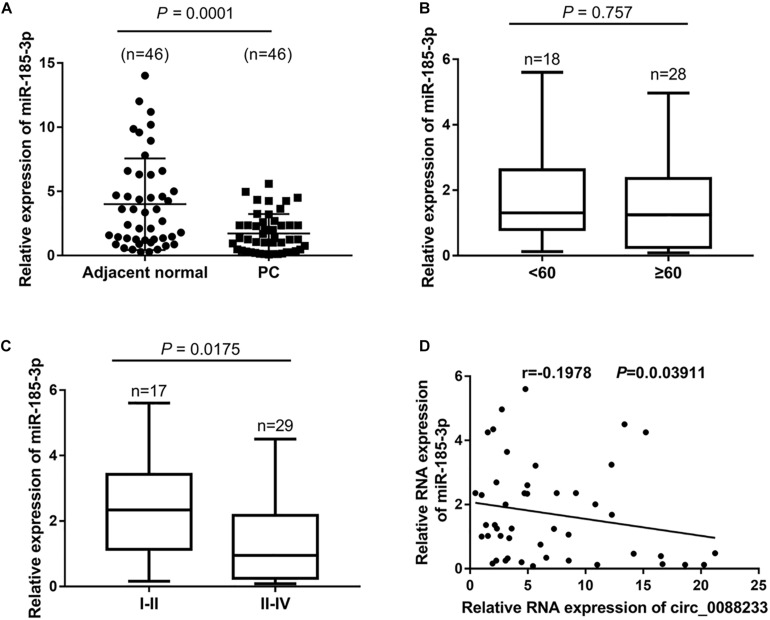
The negative correlation of the miR-185-3p level with circ_0088233 level in PC tissues. **(A)** miR-185-3p expression level in 46 prostate cancer tissues (PC) and 46 adjacent normal tissues. **(B)** Expression level of miR-185-3p in PC tissue of patients < 60 and ≥60 years of age. **(C)** Expression level of miR-185-3p in PC tissue of TNM I-II patients and TNM III-IV patients. **(D)** Negative correlation of miR-185-3p level with circ_0088233 level in PC tissues analyzed using linear regression analysis.

As shown in Figure 5A, significant downregulation of miR-185-3p was identified in PC tissues (1.25 [0.335, 2.42]) compared to adjacent normal tissues (3.02 [1.238, 6.305]). Moreover, the miR-185-3p level was not significantly different (*P* = 0.757) in the PC tissue of patients < 60 and ≥60 years of age (Figure 5B). A significant difference was evident in the PC tissue of TNM I-II patients and TNM III-IV patients (*P* = 0.0175), indicating that the miR-185-3p level was correlated with TNM stage (Figure 5C). Linear regression analysis results showed that miR-185-3p levels were negatively correlated with the circ_0088233 level in PC tissues (*r* = −0.1978, *P* = 0.03911) (Figure 5D).

### Overexpression of circ_0088233 Blocks the Effects of miR-185-3p on Cell Proliferation, Migration, Invasion, Cell Cycle, and Apoptosis

**FIGURE 6:**
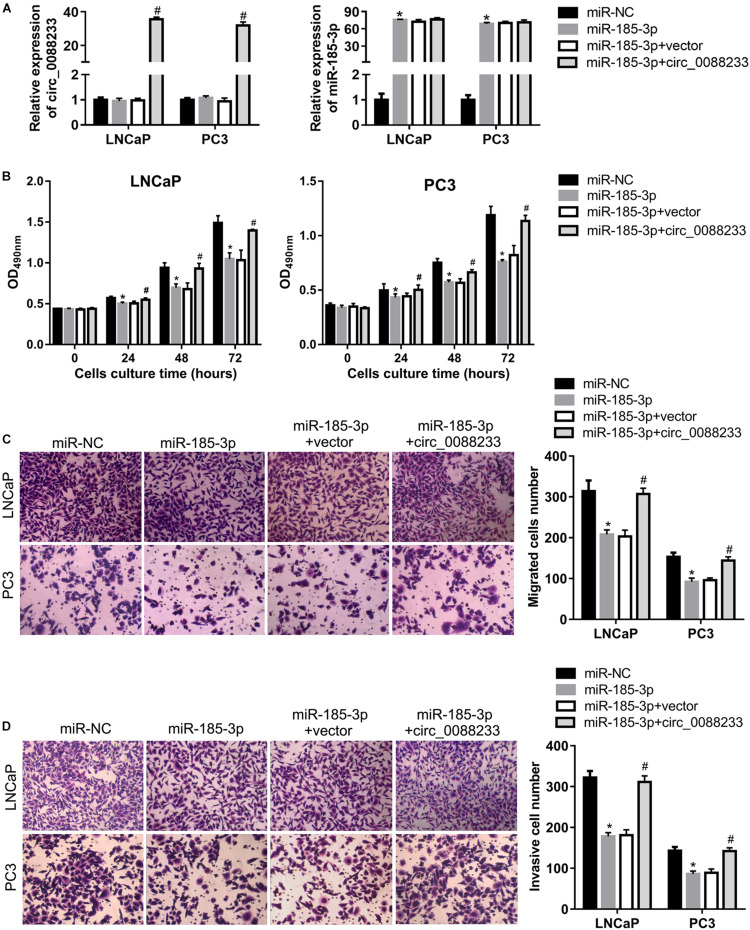
Overexpression of circ_0088233 blocks the inhibitory effects of miR-185-3p on cell proliferation, migration, and invasion. LNCaP and PC3 cells were transfected with miR-NC, miR-185-3p mimic, miR-185-3p mimic plus empty vector pLCDH-ciR (miR-185-3p + vector), or miR-185-3p mimic plus pLCDH-ciR-circ_008823 (miR-185-3p + circ_0088233). The levels of circ_0088233 and miR-185-3p were determined using qRT-PCR **(A)**. The effect of these transfections on cell proliferation **(B)**, migration **(C)**, and invasion **(D)** were evaluated by the cell proliferation assay (expressed as OD_490_), and the Transwell assay (expressed as number of migrating or invasive cells). *P < 0.05, miR-NC vs. miR-185-3p; ^#^*P* < 0.05, miR-185-3p + vector vs. miR-185-3p + circ_0088233.

**FIGURE 7:**
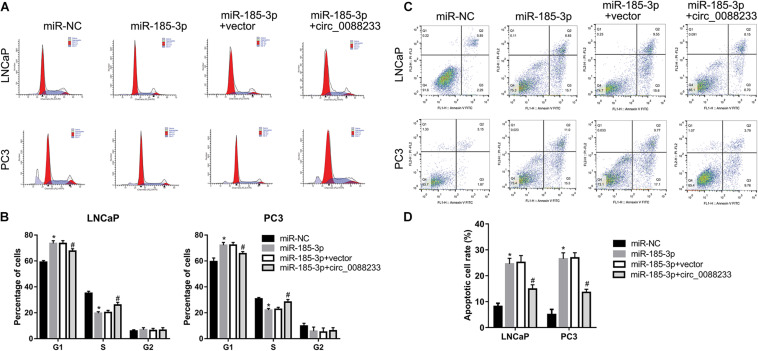
Overexpression of circ_0088233 blocks the effects of miR-185-3p on cell cycle and apoptosis. LNCaP and PC3 cells were transfected with miR-NC, miR-185-3p mimic, miR-185-3p mimic plus empty vector pLCDH-ciR (miR-185-3p + vector), or miR-185-3p mimic plus pLCDH-ciR-circ_008823 (miR-185-3p + circ_0088233). The percentage of cells in G1, S, and G2 phases **(A,B)** and apoptotic cells rate **(C,D)** were measured using flow cytometer analysis. **(A,C)** Are the representing images. **(B,D)** Are the statistical results of three independent assays. *P < 0.05, miR-NC vs. miR-185-3p; ^#^*P* < 0.05, miR-185-3p + vector vs. miR-185-3p + circ_0088233.

To investigate whether circ_0088233 plays its role through miR-185-3p, we first evaluated the effect of miR-185-3p overexpression in PC cells. As shown in Figure 6A, transfection with the miR-185-3p mimic successfully overexpressed miR-185-3p. As shown in Figure 6B, the OD_490_ of cells transfected with the miR-185-3p mimic was significantly lower than that of the miR-NC transfection group. Moreover, the number of migrating and invasive cells in the miR-185-3p mimic transfection group was significantly lower than that in the miR-NC transfection group (Figures 6C,D). These results suggested that miR-185-3p reduced the proliferation, migration, and invasion of LNCaP and PC3 cells. As shown in Figures 7A,B, the percentage of cells in the G1 phase was higher, and the percentage of cells in the S phase was lower in the miR-185-3p mimic transfection group than in the miR-NC transfection group. In addition, the percentage of apoptotic cells was higher in the miR-185-3p mimic transfection group than in the miR-NC transfection group (Figures 7C,D). These results revealed that miR-185-3p overexpression induces G1 phase arrest and apoptosis in LNCaP and PC3 cells.

To further investigate whether circ_0088233 plays its role through sponging miR-185-3p, we studied whether circ_0088233 overexpression blocks the effects of miR-185-3p overexpression. As shown in Figure 6A, circ_0088233 level increased in the miR-185-3p + circ_0088233 group compared to the miR-185-3p + vector group, indicating that pLCDH-ciR-circ_008823 could successfully overexpress circ_008823 *in vitro*. Moreover, the miR-185-3p level had no apparent change in the miR-185-3p + circ_0088233 group compared to the miR-185-3p + vector group, indicating that overexpression of circ_0088233 did not affect miR-185-3p levels. As shown in Figure 6B, the OD_490_ of the miR-185-3p + circ_0088233 group cells was significantly higher than that of the miR-185-3p + vector group. Moreover, the number of migrating and invasive cells in the miR-185-3p + circ_0088233 group was significantly higher than that in the miR-185-3p + vector group (Figures 6C,D). As shown in Figures 7A,B, the cell percentage was lower in the G1 phase, and higher in the S phase in the miR-185-3p + circ_0088233 compared to the miR-185-3p + vector group. Moreover, the percentage of apoptotic cells was lower in the miR-185-3p + circ_0088233 group than the miR-185-3p + vector group (Figures 7C,D). These results suggested that overexpression of circ_0088233 could block the effects of miR-185-3p on cell proliferation, migration, invasion, cell cycle, and apoptosis.

## Discussion

circRNAs play critical roles in modulating tumor growth, migration, and invasion of PC (Dai et al., 2018; Chen et al., 2019; Feng et al., 2019; Shen et al., 2019). Although the association between PC pathology and circRNAs remains undetermined, circRNA signatures may be useful as prognostic and predictive factors and clinical tools for assessing disease state and outcome (Kong et al., 2017; Dai et al., 2018; Huang et al., 2019; Wu et al., 2019). In this study, we screened differentially expressed circRNAs in PC using circRNA array analysis. This report is the first to identify differentially expressed circRNAs in PC. Only one matched prostate cancer, and normal tissues were used for microarray analysis in the present study. Although one sample is inadequate for microarray, this is the first step in the preliminary analysis of differentially expressed circRNAs. The expression levels of circRNAs were verified in 12 prostate cancer tissues and 12 adjacent normal tissues using qRT-PCR. Next, we further confirmed the circ_0088233 level in 46 pairs of prostate cancer and adjacent normal tissues. Therefore, we believe that one replicate microarray result did not affect our conclusion about the dysregulation of circ_0088233 in PC tissues. However, this is still a limitation.

The dysregulation of circ_0088233 in PC tissues suggested that circ_0088233 might play a role in PC. The significant correlation between the circ_0088233 level and the TNM stage further supported our hypothesis. Moreover, we investigated the biological function of circ_0088233 in PC. Our data showed that circ_0088233 knockdown reduced proliferation, migration, and invasion of PC cells, and induced G1 phase arrest and apoptosis *in vitro*. The suppressive effect of circ_0088233 knockdown on tumor growth *in vivo* was also observed in nude mice. These findings indicate that circ_0088233 may function as an oncogene in PC. To our knowledge, this is the first study to report the role of circ_0088233 in cancer, especially in PC.

Accumulating evidence indicates that circRNAs play a regulatory role in cancer by acting as miRNA sponges to abolish the inhibition effect of miRNAs on their target genes (Chen and Huang, 2018). Therefore, we further explored the molecular mechanism underlying the oncogenic function of circ_0088233 based on the ceRNA mechanism. Our results indicated that circ_0088233 could sponge miR-185-3p and function as a ceRNA of miR-185-3p. We provide a series of evidence to support this finding. First, bioinformatics analysis showed that there were two miR-185-3p binding sites in circ_0088233. Second, luciferase and biotinylated circ_0088233 probe pull-down assays revealed the direct binding of miR-183-5p to circ_0088233. Third, circ_0088233 knockdown decreased the protein level of miR-185-3p targets E2F1 and WNT2B in PC cells. Fourth, overexpression of circ_0088233 blocks the effects of miR-185-3p on cell proliferation, migration, invasion, cell cycle, and apoptosis.

Based on the ceRNA mechanism principle, we hypothesized that miR-185-3p is a tumor suppressor miRNA in PC. Our experimental results verified this hypothesis. Our findings are consistent with those of studies on NPC (Li et al., 2014; Xu et al., 2015; Liu et al., 2017) and breast cancer (Lu et al., 2018), in which miR-185-3p was considered tumor-suppressing. In PC, circ_0088233 functions as an oncogene, and miR-185-3p is a tumor suppressor miRNA. Therefore, their roles conform to the basic characteristics of the ceRNA mechanism (Guo et al., 2015; Zhang et al., 2016). Therefore, our results suggest that circ_0088233 functions as an oncogene in PC by targeting the tumor suppressor miR-185-3p. A rescue experiment, in which the overexpression of circ_0088233 blocked the effects of miR-185-3p on cell proliferation, migration, and invasion, supports this suggestion. However, there are other miRNA response elements in circ_0088233. circ_0088233 may also function as a ceRNA of other miRNAs. It is far from fully elucidated the mechanism underlying the oncogenic function of circ_0088233.

The present study has another limitation. Although we analyzed the effect of circ_0088233 knockdown on the expression of miR-185-3p targets E2F1 and WNT2B, we did not identify a direct target that can be regulated by the circ_0088233/miR-185-3p axis. Other miR-185-3p targets may also be involved in the effect of the circ_0088233/miR-185-3p axis on PC cells.

## Conclusion

We identified an upregulated circRNA, circ_0088233, in PC. Our data demonstrate that circ_0088233 regulates proliferation, migration, invasion, cell cycle, and apoptosis in PC by targeting miR-185-3p. These findings provide a better understanding of the mechanism involved in the pathogenesis of PC and will inform the development of potentially useful therapeutic strategies.

## Data Availability Statement

Publicly available datasets were analyzed in this study. This data can be found in the NCBI Gene Expression Omnibus (GSE31568).

## Ethics Statement

The studies involving human participants were reviewed and approved by Ethics Committee of Gaozhou People’s Hospital. The patients/participants provided their written informed consent to participate in this study. The animal study was reviewed and approved by Ethics Committee of Gaozhou People’s Hospital.

## Author Contributions

Z-HD, G-SY, K-LD, BP, and J-ZD contributed to the study’s conception and design. Z-HD, G-SY, and K-LD wrote the first version of the manuscript and performed the experiments. Z-HF and QH made figures. BP and J-ZD revised the final version of the manuscript. Z-HD, G-SY, and K-LD were involved in clinical data analysis. All the authors contributed to manuscript revision and read and approved the submitted version.

## Conflict of Interest

The authors declare that the research was conducted in the absence of any commercial or financial relationships that could be construed as a potential conflict of interest.

## Footnotes

1 https://www.ncbi.nlm.nih.gov/geo/query/acc.cgi?acc=GSE155792

## Supplementary Material

Supplementary Figure 1 The expression level of circ_0043592, circ_0051240, circ_0053382, circ_0088220, and circ_0088233 in 12 prostate cancer tissues and 12 adjacent normal tissues examined using qRT-PCR.

Supplementary Table 1 Primers for qRT-PCR.

Supplementary Table 2 Primers for luciferase reporter constructs.
