# Supplementary figures and images for: Hsa_circ_0088233 Alleviates Proliferation, Migration, and Invasion of Prostate Cancer by Targeting hsa-miR-185-3p
Source: Front Cell Dev Biol. 2020 Oct 30;8:528155. doi: 10.3389/fcell.2020.528155 (PMC7661849; doi:10.3389/fcell.2020.528155)

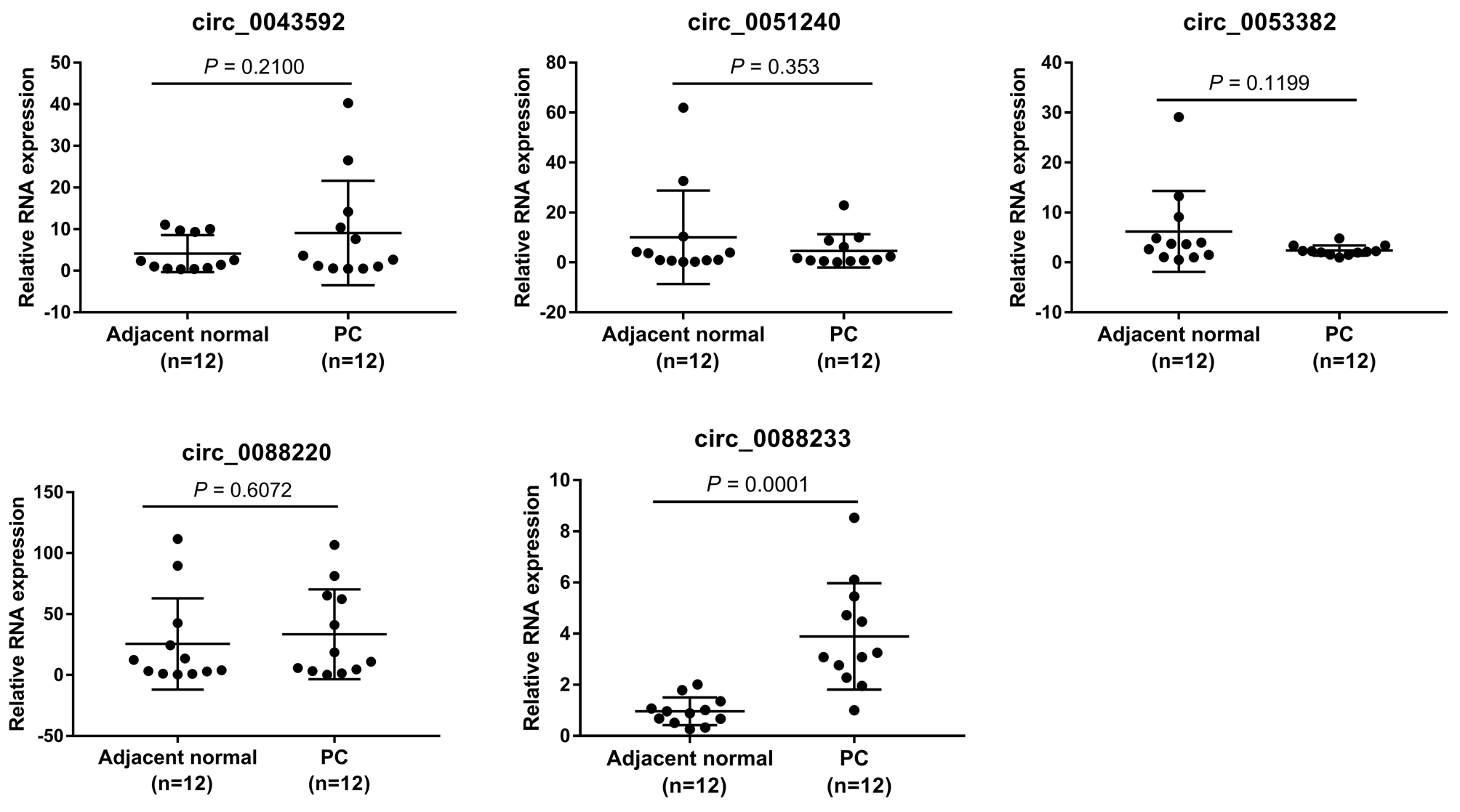

Supplement: Supplementary Figure 1 — The expression level of circ_0043592, circ_0051240, circ_0053382, circ_0088220, and circ_0088233 in 12 prostate cancer tissues and 12 adjacent normal tissues examined using qRT-PCR. [file Image_1.TIF]
