# Supplementary material for: Hsa_circ_0088233 Alleviates Proliferation, Migration, and Invasion of Prostate Cancer by Targeting hsa-miR-185-3p
Source: Front Cell Dev Biol. 2020 Oct 30;8:528155. doi: 10.3389/fcell.2020.528155 (PMC7661849; doi:10.3389/fcell.2020.528155)
Supplement: Supplementary Table 1 — Primers for qRT-PCR. [file Table_1.DOCX]

**Table S1** Primers for qRT-PCR

| **ID** | **Sequence** |
| --- | --- |
| hsa_circ_0043592-F | CCATCAGGGAAGATCATGGC |
| hsa_circ_0043592-R | CAGGTAGGCTAGCTCCTCAT |
| hsa_circ_0051240-F | TGGCCGCAATAATTCCATAGT |
| hsa_circ_0051240-R | GTGTCCGGCCCATCAGTC |
| hsa_circ_0053382-F | TTGCCCAAGGTGAAATGCTT |
| hsa_circ_0053382-R | GCAGTGTGCATCTCTTTGAC |
| hsa_circ_0088220-F | GTGCAGTATGCTTCCAACGC |
| hsa_circ_0088220-R | CAACAGGCGCTGGTTTCCTG |
| hsa_circ_0088233-F | TCGGAAGTCAAAGAAAGACCA |
| hsa_circ_0088233-R | ATCACCTTGGTTCGACACAC |
| GAPDH-F | GGGAAACTGTGGCGTGAT |
| GAPDH-R | GAGTGGGTGTCGCTGTTGA |
| hsa-miR-185-3p-F | ACACTCCAGCTGGGGACCAGAGGAAAGCCAGC |
| hsa-miR-185-3p-R | CTCAACTGGTGTCGTGGA |
| hsa-miR-185-3p-RT | CTCAACTGGTGTCGTGGAGTCGGCAATTCAGTTGAGaggggctg |
| U6-F | GGGAAACTGTGGCGTGAT |
| U6-R | GAGTGGGTGTCGCTGTTGA |

F: forward primer; R: reverse primer; RT: Primer for reverse transcription.
