# Supplementary material for: Hsa_circ_0088233 Alleviates Proliferation, Migration, and Invasion of Prostate Cancer by Targeting hsa-miR-185-3p
Source: Front Cell Dev Biol. 2020 Oct 30;8:528155. doi: 10.3389/fcell.2020.528155 (PMC7661849; doi:10.3389/fcell.2020.528155)
Supplement: Supplementary Table 2 — Primers for luciferase reporter constructs. [file Table_2.DOCX]

**Table S2** Primers for luciferase reporter constructs

| ID | Primer sequence |
| --- | --- |
| hsa_circ_0088233-XhoI-F | 5'ccgctcgagAGACCAAGGTGAACAATGCGACGACA |
| hsa_circ_0088233-NotI-R | 5'ataagaatgcggccgcTTCTTTGACTTCCGAGAGGATCCAGG |
| Mutant 1-hsa_circ_0088233-F | 5'GATTATCCCTGTGGTTTCGACTTGTCATTCATTTGTCTACCACAGCCAGGCGGTACGTGT 3' |
| Mutant 1-hsa_circ_0088233-R | 5'GTAGACAAATGAATGACAAGTCGAAACCACAGGGATAATCAGGGGATTGTTCCTGCAGCT 3' |
| Mutant 2-hsa_circ_0088233-F | 5'GCTGCCAGAGCAAAACACTTGCTCATTTGGCCGAGCAGAGCTGCGTGCACTTCGCATGTG |
| Mutant 2-hsa_circ_0088233-R | 5'GCTCGGCCAAATGAGCAAGTGTTTTGCTCTGGCAGCTGCTCAGGGTGACGGGGTCAAAGT |

Mut: mutant; F: forward primer; R: reverse primer.
